# Characterization of purified and Xerogel immobilized Novel Lignin Peroxidase produced from *Trametes versicolor* IBL-04 using solid state medium of Corncobs

**DOI:** 10.1186/1472-6750-12-46

**Published:** 2012-08-03

**Authors:** Muhammad Asgher, Hafiz Muhammad Nasir Iqbal, Muhammad Irshad

**Affiliations:** 1 Industrial Biotechnology Laboratory, Department of Chemistry and Biochemistry, University of Agriculture, Faisalabad, Pakistan; 2 Department of Biochemistry, Nawaz Sharif Medical College, University of Gujrat, Gujrat, Pakistan

**Keywords:** *T. versicolor* IBL-04, LiP, Immobilization, Xerogel, Characterization, Hyper-activation, Thermo-stabilization, Inactivation tolerance

## Abstract

**Background:**

Cost-effective production of industrially important enzymes is a key for their successful exploitation on industrial scale. Keeping in view the extensive industrial applications of lignin peroxidase (LiP), this study was performed to purify and characterize the LiP from an indigenous strain of *Trametes versicolor* IBL-04. Xerogel matrix enzyme immobilization technique was applied to improve the kinetic and thermo-stability characteristics of LiP to fulfil the requirements of the modern enzyme consumer sector of biotechnology.

**Results:**

A novel LiP was isolated from an indigenous *T. versicolor* IBL-04 strain. *T. versicolor* IBL-04 was cultured in solid state fermentation (SSF) medium of corn cobs and maximum LiP activity of 592 ± 6 U/mL was recorded after five days of incubation under optimum culture conditions. The crude LiP was 3.3-fold purified with specific activity of 553 U/mg after passing through the DEAE-cellulose and Sephadex-G-100 chromatography columns. The purified LiP exhibited a relatively low molecular weight (30 kDa) homogenous single band on native and SDS-PAGE. The LiP was immobilized by entrapping in xerogel matrix of trimethoxysilane (TMOS) and proplytetramethoxysilane (PTMS) and maximum immobilization efficiency of 88.6% was achieved. The free and immobilized LiPs were characterized and the results showed that the free and immobilized LiPs had optimum pH 6 and 5 while optimum temperatures were 60°C and 80°C, respectively. Immobilization was found to enhance the activity and thermo-stability potential of LiP significantly and immobilized LiP remained stable over broad pH and temperature range as compare to free enzyme. Kinetic constants *K*_m_ and *V*_max_ were 70 and 56 μM and 588 and 417 U/mg for the free and immobilized LiPs, respectively. Activity of this novel extra thermo-stable LiP was stimulated to variable extents by Cu^2+^, Mn^2+^ and Fe^2+^ whereas, Cystein, EDTA and Ag^+^ showed inhibitory effects.

**Conclusions:**

The indigenously isolated white rot fungal strain *T. versicolor* IBL-04 showed tremendous potential for LiP synthesis in SSF of corncobs in high titters (592 U/mL) than other reported *Trametes* (*Coriolus*, *Polyporus*) species. The results obtained after dual phase characterization suggested xerogel matrix entrapment a promising tool for enzyme immobilization, hyper-activation and stabilization against high temperature and inactivating agents. The pH and temperature optima, extra thermo-stability features and kinetic characteristics of this novel LiP of *T. versicolor* IBL-04 make it a versatile enzyme for various industrial and biotechnological applications.

## Background

Fungi from basidiomycetes group are known ligninolytic enzymes producers. Lignin modifying enzymes (LMEs) are rarely produced by bacteria, yeasts and most fungi but frequently occur in the fermented culture broth of white rot fungi [1,2]. White rot fungi (WRF) are so far exclusive in their potential to entirely degrade all the components of lignocellulosic materials and this capability is due to their extra cellular nonspecific LMEs which function together with H_2_O_2_ and secondary metabolites. The aptitude of *Trametes versicolor* to depolymerize lignin has been investigated from a physiological point of view. In *P. chrysosporium* and *T. versicolor* lignin peroxidases have been found to appear during the secondary metabolism [1,3,4]. Lignin peroxidases (LiPs) are glycosylated proteins that functionally require H_2_O_2_ for the oxidation of lignin related aromatic structures. A large number of substrates and by-products of lignin degradation, such as vanillic acid, chlorogenic acid, veratric acid, and veratryl alcohol, have been tested for their ability to boost up ligninase activity [5]. Veratryl alcohol a secondary metabolite produced by ligninolytic WRF plays an important role in LiP catalysis. The kinetic analysis has revealed that cationic radical of veratryl alcohol converts LiP (II) and/or LiP (III) to LiP and improves its catalytic cycle [6]. LiPs from various WRF including *Trametes versicolor* and *Pleurotus ostreatu* differ from the other oxidoreductases in that they have low pH optima varying between pH 2–5 and much higher redox potentials [7].

LiP is a biotechnologically important enzyme having potential applications to degrade highly toxic phenolic compounds from bleach plant effluents. LiPs and other ligninolytic enzymes from WRF find numerous applications in various industrial processes such as degradation of dyes, bioremediation, delignification for ethanol production, oxidation of organic pollutants, biosensors development, textile bio-finishing, beverage processing, wastewater detoxification, denim stone washing and detergent manufacturing [1,2,8-12]. Significant efforts have been made to convert lignocellulosic residues to valuable products such as bio-fuels, chemicals and animal feed with the help of ligninolytic enzymes (LiP, MnP and laccase) of WRF, many of which have been successful.

Enzyme immobilization has revolutionized the field of enzyme biotechnology. Entrapment, adsorption and surface binding are the most frequently employed methods that have been applied recently for enzyme immobilization. Entrapment is preferred over surface binding as this method is easier and cheaper and the structure of the enzyme remains secure [13]. Recently, the physical characteristics of xerogels have been manipulated for enzyme immobilization. Hydrophobic xerogels have the ability to produce enzymes in defined thin films that are thermo-stable and have the potential to catalyze reactions under wide environmental conditions [3]. Moreover, xerogel polymers are non-toxic and do not swell in aqueous or organic solvents, thus preventing the leaching of entrapped enzyme and allowing the enzymes to maintain their native structures. Immobilization of enzymes presents additional advantages including, improved resistance to thermal and chemical inactivation and remarkable storage and operational stability. In spite of the clear advantages of enzyme immobilization, only about 20% of bio-catalytic processes involve immobilized enzymes. However, over the last few years a number of interesting achievements and patent applications have been reported [14], indicating that enzyme immobilization has entered into a thrilling new stage.

*T. versicolor* is one of the most potent lignin degrading microorganisms that produce extracellular peroxidases under optimum growth conditions [5]. The occurrence of the ligninolytic enzymes in the *T. versicolor* genome makes it an attractive fungus for miscellaneous biotechnological and environmental applications. In our previous studies [2,4,15,16], we have successfully investigated the extracellular ligninolytic enzymes (MnP, LiP and laccase) synthesis potential of different locally isolated indigenous WRF strains including *Trametes versicolor* IBL-04 under solid and liquid state fermentation based on varying lignocellulosic substrates and dye decolorization. Although considerable work has been reported on LiP from different fungal species but there are very few reports on purification, characterization and immobilization of LiP produced from *Trametes versicolor*. In this study for the first time *T. versicolor* IBL-04 LiP was immobilized by xerogel entrapment method with an objective of dual phase characterization of novel thermo-stable LiP to investigate its potential for industrial applications.

## Results and discussion

### Production of LiP

*T. versicolor* IBL-04 was cultivated on solid substrate fermentation medium of corncobs moistened (60% w/w moisture) with Kirk’s basal medium of pH 4.0 and incubated at 30°C for five days under previously optimized SSF growth conditions [4]. Maximum LiP activity of 592 ± 6 U/mL was recorded when corncobs supplemented with glucose and yeast extract in 25:1 C/N ratio, 1 mL of 1% tween-80 as surface active agent, and ZnSO_4_ as metal ion source was inoculated with 5 mL freshly prepared spore suspension of *T. versicolor* IBL-04 and fermented for 5 days under still culture SSF conditions at 30°C. Extracellular ligninolytic enzymes production is strongly influenced by the nature and amount of nutrients and microelements in the growth substrate. Different WRF have particular responses to nutrients and show different growth and enzyme synthesis patterns during their growth on different substrates [17]. *T. versicolor* IBL-04 had an extraordinary potential to produced relatively higher amount of novel thermo-stable LiP through SSF of corncobs than those described in literature for different strains of *Trametes versicolor, Cunninghamella elegans* and *Flavodon flavus*[18-21].

### Purification of LiP

The cell free crude enzyme extract had an initial LiP activity of 118400 U/200 mL and specific activity of 170 U/mg. LiP was maximally precipitated out at 80% ammonium sulphate saturation with specific activity of 248 U/mg and 1.5-fold purification. By Sephadex G-100 column gel filtration chromatography, the enzyme was purified up to 2.6-fold with specific activity of 440U/mg and its purification increased to 3.3-fold with specific activity of 553 U/mg after passing through the DEAE-Cellulose ion exchange column (Table 1). Mtui and Nakamura [19] achieved 50-80% (NH_4_)_2_SO_4_ saturation, followed by chromatographic purification techniques for the recovery of pure LiP. Roushdy et al. [18] achieved 2.76 purification fold with specific activity of 119.34 U/mg using Sephadex G-100 gel permeation of LiP after 80% ammonium sulfate saturation.

**Table 1:** **Purification summary of LiP produced by *T. versicolor* IBL-04**

**Purification steps**	**Total volume (mL)**	**Total enzyme activity (U)**	**Total Protein content (mg)**	**Specific activity (U/mg)**	**Purification fold**	**% Yield**
Crude enzyme	200	118400	698	170	1	100
(NH_4_)_2_SO_4_ precipitation	25	13375	54	248	1.5	11.3
Sephadex-G-100	12	5724	13	440	2.6	4.8
DEAE-Cellulose	9	3870	7	553	3.3	3.2

### Molecular mass estimation by electrophoresis

**Figure 1:**
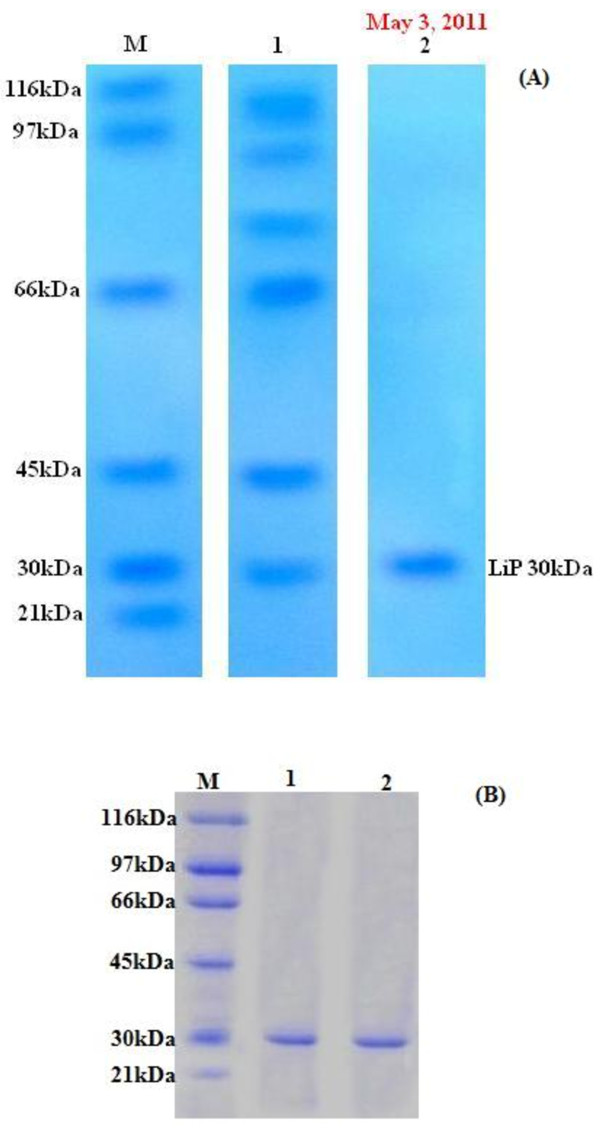
**SDS (A) and Native (B) PAGE of LiP isolated from *T. versicolor * IBL-04.**

The purified LiP was resolved on native and SDS-PAGE and found to be a homogenous monomeric protein, as evident by single homogenous band corresponding to 30 kDa (Figure 1). The *T. versicolor* IBL-04 LiP was different in molecular mass from previously reported LiPs from *Cunninghamella elegans* and *Phanerochaete sordida* YK-624 (50 kDa) [18,22], *Hexagona tenuis* MTCC 1119 (48 kDa) [23], *Flavodon flavus* (46 kDa) [19], *Loweporus lividus* MTCC-1178 (40 kDa) [24] and *Trametes versicolor* (40 kDa) [25].

[Lane M, Molecular weights in kDa of standard marker; (β-Galactosidase, 116 kDa; Phosphorylase B, 97 kDa; albumin, 66 kDa; ovalbumin, 45 kDa; carbonic anhydrase, 30 kDa and trypsin inhibitor, 21 kDa); lane 1, Crude enzyme extract lane 2, Purified LiP in SDS; lane 1 and 2 in native, Purified LiP (30 kDa)].

### Immobilization of LiP

The activity profiles of free and immobilized LiPs showed that specific activities of xerogel immobilized LiP was 1.27 fold higher as compared to free enzyme as shown in Table 2. The influence of enzyme concentration on the immobilization efficiency was studied using enzyme in the range of 2–10 mg/mL (563 U/mg). The fraction containing 2 mg/mL enzyme (563 U/mg) concentration showed maximum immobilization efficiency (88.6%). The immobilization efficiency of gel matrix decreased to 51.2% with increasing concentrations of enzyme (Table 2). Entrapment of LiP in xerogel matrix involves adsorption phenomenon that has been reported as the best method for immobilization of enzymes [3]. Previously, entrapment of LiP from *P*. *chrysosporium* in xerogels caused hyperactivation but an increase in hydrophobic character above certain optimum limits caused a decrease in LiP activity [3]. The results obtained by the xerogel entrapment method for the present LiP immobilization are comparable to those obtained by other methods such as the covalent bonding of the enzyme on siliceous cellular foams (McFs), Sepabeads EC-EP3 and Dilbeads NK supports or immobilization by the formation of cross-linked enzyme aggregates [9,26]. However, the covalent binding strategy adopted in the above mentioned studies was much more expensive as compare to the xerogel matrix entrapment method due to the requirement of a coupling agent such as glutareldehyde.

**Table 2:** Specific activities* of free and immobilized LiP with immobilization efficiency^**^

**LiP concentration (mg/mL)**	**Specific activity of free LiP (U/mg)**	**Specific activity of immobilized LiP (U/mg)**	**Immobilization efficiency (%)**
2	580	666	88.6
4	598	790	82.2
6	644	865	73.4
8	702	933	62.6
10	788	999	51.2

*Specific activities were calculated in U/mg of triplicate means.

**Immobilization efficiency was calculated as the ratio of the enzyme entrapped (difference between the enzyme loaded and the enzyme in the supernatant after washing) × 100.

### Characterization of free and immobilized LiP

#### Effect of pH on LiP activity and stability

**Figure 2:**
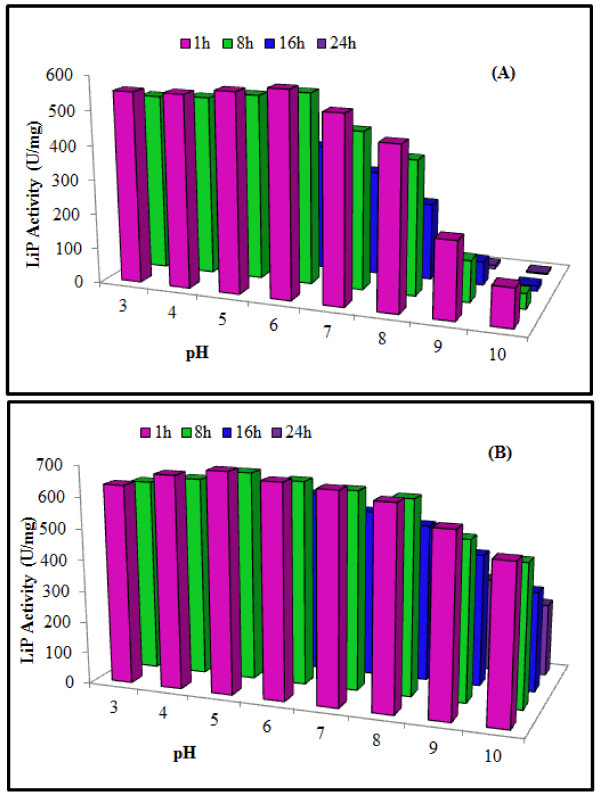
**Effect of different pH on the activity and stability of purified free (A) and xerogel matrix immobilized (B) *T. versicolor * IBL-04 LiP.**

The pH-activity profile of free LiP displayed optimal activity at pH 6 whereas, immobilization hyper-activated the LiP and slightly shifted pH optima towards more acidic pH range. Immobilization also enhanced the resistance of LiP against pH variation. Results of stability profile showed that free LiP was only stable for 1 h in a pH range of 3.0 to 6.0 and start loosing activity at higher pH values steadily. The xerogel entrapment enhanced the pH stability of LiP for longer time period of 24 h that was much higher than the free enzyme (Figure 2). Earlier studies [7,27] reported optimum activities of various WRF LiPs to vary between pH 2–5. [Alam et al. 28] reported that the LiP produced from *P*. *chrysosporium* showed more than 80% of the maximum activity at optimum pH 5, while according to [Rodríguez-Couto and Sanroman 29] LiP from *P*. *chrysosporium* was optimally active at pH 4.2. It was observed that after immobilization, the optimum pH of LiP was shifted slightly toward the acidic range possibly due to the buffering effect of the carrier surface. This phenomenon was also reported by [Erdemir et al. 30], who observed a slight optimum pH shift from 7 to 6 for LiP due to immobilization, but this smaller difference was due to the different method of immobilization, as well as the nature of the supporting matrix that was calyx[n]arene.

#### Effect of temperature on LiP activity and stability

**Figure 3:**
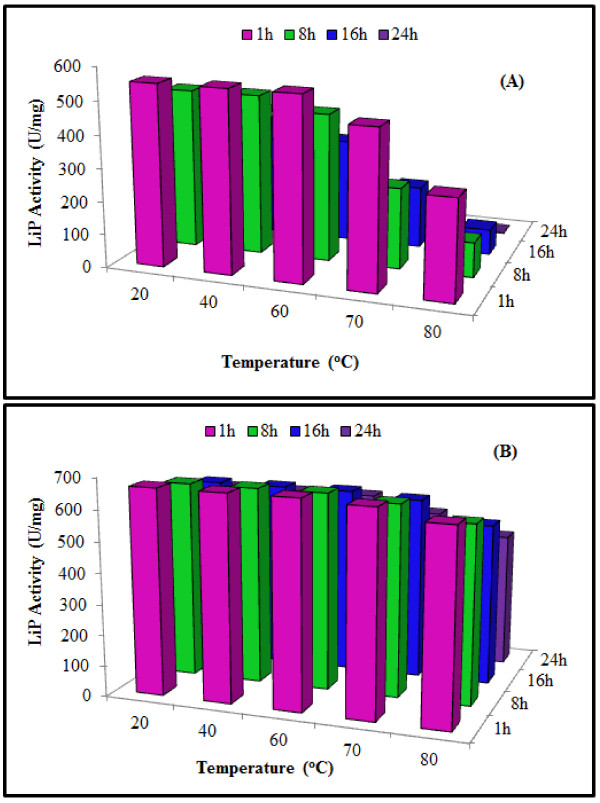
**Effect of different temperatures on the activity and stability of purified free (A) and xerogel matrix immobilized (B) *T. versicolor * IBL-04 LiP.**

The free LiP from *T. versicolor* IBL-04 was optimally active at 60°C and further increase in temperature caused its deactivation, while xerogel entrapped LiP showed optimum activity at temperatures higher than 60°C. As compared to free enzyme xerogel entrapped LiP had an extraordinary thermo-stability for up to 24 h incubation at 80°C without losing much of its activity (Figure 3). As compared to previously reported LiPs the *T. versicolor* IBL-04 LiP had greater thermo-stability suggesting its potential for biotechnological applications. Relatively greater activity and high thermo-stability are attractive and desirable characteristics of an enzyme for industrial applications [15]. LiP from *Loweporus lividus* MTCC-1178 was optimally active at 24°C [24], LiP from *P*. *chrysosporium* showed better thermo-stability and was optimally active at 55°C [28], and that from *P*. *chrysosporium* was stable at 34°C [29]. Immobilization to a solid support causes changes in the enzyme conformation and thus increases stability towards heat denaturation. Similar hyper-thermostablization of other enzymes in hydrophobic xerogels has also previously been reported [3].

#### Effect of substrate concentration: Determination of K_m_ and V_max_

**Figure 4:**
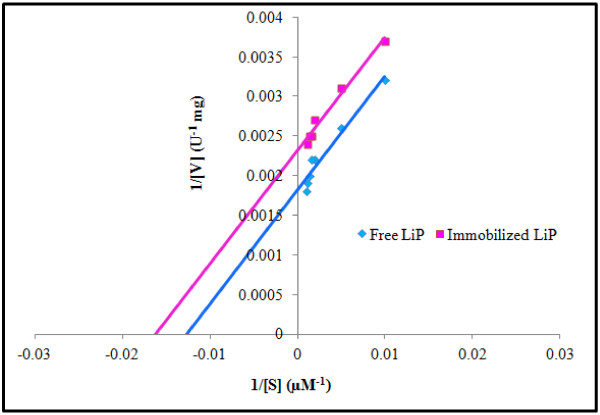
**Lineweaver-Burk reciprocal plot: Determination of *K* **_**m **_**and *V* **_**max **_**for free and immobilized LiP.**

Varying concentrations of veratryl alcohol (μM) were plotted against the respective initial specific activities (V_o_) of free and immobilized LiP. Michalis-Menten kinetics yielded a hyperbolic curve. Lineweaver-Burk double reciprocal plots (Figure 4) were constructed. The *K*_m_ and *V*_max_ values for free LiP were 70 μM and 417 U/mg as compared to 56 μM and 588 U/mg, respectively for immobilized LiP. The lower value of *K*_m_ and higher V_max_ values for immobilized enzyme indicated that immobilization enhanced the substrate affinity and catalytic efficiency of LiP. [Asgher et al. 3] reported that immobilized enzymes have the best catalyzing power as well as good interaction for their substrate. There is slight difference in the *K*_m_ and *V*_max_ values for *T. versicolor* IBL-04 LiP than other reported LiPs. The *K*_m_ values for veratryl alcohol and H_2_O_2_ for LiP from *Loweporus lividus* MTCC-1178 were 58 and 83 mM, respectively [24]. In another study the *K*_m_ for LiP was 167 μM using veratryl alcohol as substrate [31].

#### Effect of activators/inhibitors on free and immobilized LiP

**Figure 5:**
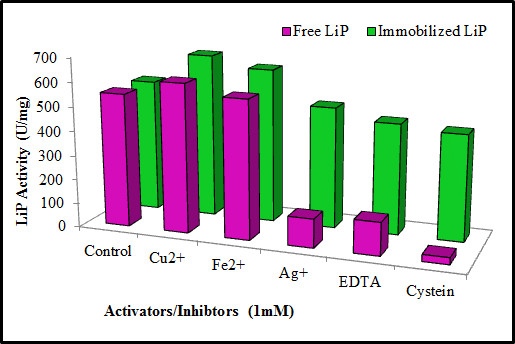
Effect of different activators/inhibitors on free and immobilized LiP.

The stimulatory/inhibitory effects of different organic compounds (EDTA & Cystein) and metal ions (Cu^2+^, Fe^2+^ & Ag^+^) on free and entrapped LiP was investigated. Results showed that LiP was completely inhibited by cystein while it was partially inhibited with EDTA. Among the metal ions used, only Ag^+^ caused LiP inhibition to variable extent whereas, Cu^2+^ and Fe^2+^ were activators of LiP at low concentration whereas (Figure 5). The xerogel entrapped LiP was found to exhibit significant tolerance against inactivation by cystein, EDTA and Ag^+^. EDTA is a metal chelating agent that has ability to bind the inorganic prosthetic groups of enzymes leading to inhibitory effect on enzymes. In many previous studies metal ions like Hg^2+^ and Ag^+^ have been reported as stronger inhibitors of extra cellular peroxidases of WRF, while Fe^3+^, Ca^2+^ and Ni^2+^ did not cause any alteration in LiP activity [2]. Low concentrations of heavy metals ions are necessary for the development of the ligninolytic enzyme system of various WRF. Addition of Zn^2+^ and Cu^2+^ into the cultivation medium at low concentrations has been reported to increase LiP activity [15].

## Conclusions

In conclusion, *T. versicolor* IBL-04 showed tremendous potential for LiP synthesis in SSF of corncobs in high titters (592 U/mL), than previously reported *Trametes* species. The extracellular LiP from *T. versicolor* IBL-04 had lowered molecular as compare to most WRF LiPs. *T. versicolor* IBL-04 LiP successfully immobilized with immobilization efficiency of 88.6% by xerogel entrapment in a matrix of TMOS and PTMS. Dual phase characterization showed that immobilization enhanced LiP activity, stability over broad pH range (3–8) and thermotolerance in 20-80°C temperature range for extended time period of 24 h. Immobilization also enhanced the substrate affinity and catalytic efficiency of LiP as evidenced by lower *K*_m_ and high *V*_max_ values. The xerogel entrapped LiP had significantly higher tolerance against inactivating agents as compared to free enzyme. Results of dual phase characterization suggested that xerogel matrix entrapment is a promising tool for hyper-activation and stabilization of LiP making it a valuable and versatile enzyme for various industrial and biotechnological applications.

## Methods

### Chemicals and lignocellulosic substrate

Trimethoxysilane, proplytetramethoxysilane, polyvinyl alcohol, Sephadex G-100, 2, 2′-azino-bis-3-ethylbenzothiazoline-6-sulfonic acid (ABTS), Coomassie Brilliant Blue G-250, sodium dodecylsulphate (SDS), trizma base and standard Protein markers were purchased from Sigma-Fluka-Aldrich (USA). All other chemicals were of analytical grade and were mainly purchased from Merck (Germany) and Scharlau (Spain). Corncobs used a substrate in SSF was collected from CPC-Rafhan, Faisalabad, Pakistan. The substrate was chopped, oven dried (60°C), ground to fine particle size (40 mm) and stored in air tight plastic jars to avoid moisture. The substrate was used in SSF without any pretreatment.

### Micro-organism and preparation aqueous spore inoculum

The indigenous strain *T. versicolor* IBL-04 available in Industrial Biotechnology Laboratory, UAF, Pakistan was used for LiP production. Aqueous spore suspension was prepared by growing the *T. versicolor* IBL-04 in glucose (1%) supplemented Kirk’s basal nutrient medium [32]. The medium was sterilized (121°C) in laboratory scale autoclave (Sanyo, Japan) for 15 min. It was allowed to cool and was inoculated with loopful culture of *T. versicolor* IBL-04 from PDA slants in laminar air flow (Dalton, Japan). The inoculated flask was incubated for 5 days at 30°C in an orbital shaker (120 rpm) (Sanyo-Gallemkemp, UK) to get homogenous spore suspension (10^6^ -10^8^ spores/mL).

### LiP production and extraction protocol

Production of LiP from *T. versicolor* IBL-04 was carried out in 250 mL Erlenmeyer flasks under some previously optimized fermentation conditions [4]. Triplicate flasks containing 5 g corncobs were moistened (60% w/w) with Kirk’s nutrient medium, sterilized and inoculated with 5 mL of freshly prepared homogeneous fungal spore suspension under sterile conditions in laminar air flow. The inoculated flasks were allowed to ferment at 30°C in a still culture SSF incubator (EYLA SLI-600ND, Japan) for 5 days. After the expiry of 5 days fermentation time, the LiP was extracted by adding 100 mL distilled water to the fermented mesh and flasks were shaken at 120 rpm for 30 min. The contents of fermentation flask were filtered (Whatman No.1 filter paper) and washed thrice with distilled water. The filtrates were centrifuged at 4,000 × g for 10 min and clear supernatants were pooled as crude extract for LiP assay and purification studies.

### LiP activity assay and protein content determination

LiP activity of the supernatant was determined by the method of [Tien and Kirk 32] following the H_2_O_2_ dependent oxidation of veratryl alcohol to verataldehyde at 25°C using UV/Visible spectrophotometer (T60, PG Instruments, UK). The assay mixture contained 1 mL tartarate buffer (100 mM) of pH 3, 1 mL of 4 mM veratryl alcohol, 500 μL of 0.2 M H_2_O_2_ and 100 μL of enzyme extract. The activity of reaction mixture was measured against reagent blank at 310 nm (**ϵ**_310_ = 9300). The recorded activities were expressed as U/mL while one unit LiP activity was defined as the amount of enzyme required to oxidize one μmol of VA per minute at 25°C. To determine the amount of protein contents of the crude and purified enzyme extracts, the Bradford micro assay was followed [33] using Bovine serum albumin (BSA) as standard.

### Purification of LiP

Crude LiP extract was centrifuged at 4,000 × g for 15 min and cell-free supernatant was first brought to 40% saturation by the gradual addition of solid crystals of ammonium sulfate and kept for overnight at 4°C. The resulting precipitate was collected by centrifugation (4,000 × g) for 15 min at 4°C and in the supernatant more crystals of ammonium sulfate were added in order to achieve 80% saturation. It was again kept for overnight at 4°C and centrifuged as described previously. After centrifugation the sediments were dissolved in a minimal volume of 100 mM Tartarate buffer of pH 3. The solution was kept in a dialysis bag and after sealing securely it was dialyzed against the same buffer. The dialyzate was concentrated by ultra-filtration and applied to Sephadex G-100 (Sigma, USA) glass column (2 × 20 cm). Tartarate buffer (100 mM) with 0.15 M NaCl was used as elution buffer and the flow rate was maintained at 0.5 mL min^-1^. Up to 25 LiP positive fractions were pooled, concentrated by ultra filtration and used to determine the enzyme activity as well as the protein content as described earlier. For further purification, the fractions collected after passing through Sephadex G-100 chromatographic column were loaded on DEAE cellulose ion-exchange column (2 × 20 cm). The fractions were collected with flow rate of 1 mL per min and both the LiP activity and protein contents were determined for each fraction as mentioned in the previous section.

### Molecular mass estimation by electrophoresis

To determine the relative molecular mass of purified LiP, sodium dodecyl sulphate (SDS) and Native poly acrylamide gel electrophoresis (PAGE) was performed on a stacking and separating gel according to the method of [Laemmli 34] using Mini-gel electrophoresis (V-GES, Wealtec, USA). The molecular mass of the purified LiP was estimated in comparison to standard molecular weight markers (standard protein markers, 21–116 kDa; Sigma, USA). The protein bands were visualized by staining with Coomassie Brilliant Blue G-250 (Sigma, USA) after documentation.

### Immobilization of LiP

The trimethoxysilane (TMOS) and proplytetramethoxysilane (PTMS) were used in molar TMOS: PTMS (T: P) ratios of 1:5 to prepare xerogel matrix for LiP entrapment. *Trametes versicolor* IBL-04 LiP was suspended in de-ionized water in five different fractions (2-10 mg/mL) having 563 U/mg LiP activity and centrifuged to remove insoluble components. The supernatant fluid (400μL) from each fraction was added to a mixture of aqueous sodium fluoride, polyvinyl alcohol and water. The solution was shaken and PTMS was added followed by TMOS. The reaction mixture was shaken for 20 sec on vortex mixer and placed in ice bath until gel formation occurred. At the end activity of xerogel entrapped LiP was determined, as described in the previous section for free enzyme.

### Characterization of free and immobilized LiP

The kinetic and catalytic properties of free and immobilized LiP were studied through characterization by studying the effect of pH, temperature, substrate concentration and activators/inhibitors on their activities.

#### Effect of pH on LiP activity and stability

The effect of varying pH (pH 3–10) on the activities of free and entrapped LiP was investigated using the following buffers (0.2 M): sodium melonate buffer, pH 3.0 & 4.0; citrate phosphate, pH 5.0 & 6.0; sodium phosphate, pH 7.0 & pH 8.0; and potassium carbonate buffer, pH 9.0 & pH 10.0. The free and gel entrapped LiPs were incubated at room temperature (25°C) for 15 min. While, for stability assay, LiP was incubated at room temperature (25°C) for up to 24 h without substrate before running the normal activity assay and residual activity of LiP was checked after 1, 8, 16 and 24 h.

#### Effect of temperature on LiP activity and stability

Effect of varying temperatures on LiP activity and stability was also studied. Both the free and xerogel entrapped LiP were incubated with citrate phosphate buffer of pH 6.0 and 5.0, respectively, for 15 min at varying temperatures (20-80°C) before running the enzyme assay. For stability studies, the enzymes were incubated at varying temperatures for 24 h in the absence of substrate before running the normal activity assay and residual enzyme activities were determined after 1, 8, 16 and 24 h.

#### Effect of substrate concentration: Determination of K_m_ and V_max_

The Michalis-Menten kinetic constants *K*_m_ and *V*_max_ for free and entrapped LiP were determined from Michalis-Menten and Lineweaver–Burk reciprocal plots using varying concentrations of veratryl alcohol (100–1000 μM) as substrate. To determine the kinetic constants free and xerogel entrapped LiP were incubated for 15 min at room temperature (25°C) using citrate phosphate buffer of pH 6.0 and 5.0, respectively, before carrying out enzyme assay.

#### Effect of activators/inhibitors on free and immobilized LiP

Effect of various organic (EDTA & Cystein) and inorganic ions (Cu^2+^, Fe^2+^& Ag^+^) as possible stimulators or inhibitors on purified free and xerogel entrapped LiP from *T. versicolor* IBL-04 was studied. The enzyme activities in each case were determined under standard assay conditions as described earlier (incubation time 15 min at 25°C in citrate phosphate buffer of pH 6.0 and 5.0 for free and xerogel entrapped LiP, respectively).

### Statistical analysis

All the experiments and analyses were performed in triplicate and the data were statistically evaluated according to [Steel et al. 35].The means and standard errors of means (Mean ± S.E) were computed for each treatment and S.E values have been displayed as Y-error bars in figures.

## Competing interests

The authors have no competing interests.

## Authors’ contributions

HMNI (Research Associate of the project) participated in designing of the study and carried out the experimental work on microbial cultivation, LiP production & extraction, purification, xerogel entrapment immobilization and kinetic characterization of free and immobilized LiP and participated in drafting the manuscript. All the research work was carried out under supervision of MA (Principal Investigator of the project) who designed and coordinated the experiments. MA and MI also interpreted the data and drafted the manuscript. All authors read and approved the revised manuscript.
